# Evaluating the sustainability of indirect potable reuse and direct potable reuse: a southern Nevada case study

**DOI:** 10.1002/aws2.1153

**Published:** 2019-08-27

**Authors:** Cory Dow, Sajjad Ahmad, Krystyna Stave, Daniel Gerrity

**Affiliations:** ^1^ Department of Civil and Environmental Engineering and Construction University of Nevada Las Vegas Nevada; ^2^ Carollo Engineers Las Vegas Nevada; ^3^ School of Public Policy and Leadership University of Nevada Las Vegas Nevada; ^4^ Applied Research and Development Center Southern Nevada Water Authority Las Vegas Nevada

**Keywords:** direct potable reuse, indirect potable reuse, net present worth, sustainability, system dynamics, water supply

## Abstract

This case study presents a framework for evaluating the sustainability of indirect potable reuse (IPR) and direct potable reuse (DPR) in Las Vegas, Nevada. A system dynamics model was developed to simulate population growth, water supply, water quality, energy costs, net present worth (NPW), and greenhouse gas (GHG) emissions. The model confirmed that DPR could achieve a net reduction in energy costs of up to US$250 million while still ensuring an adequate water supply. However, the high NPW of DPR ($1.0–$4.0 billion) relative to the status quo IPR approach ($0.6 billion) represents a significant economic hurdle, although future monetization of salt loadings and GHGs could reduce that disparity. DPR with ozone‐biofiltration would also be hindered by an estimated concentration of total dissolved solids of up to 1,300 mg/L. Despite these barriers to implementation in Las Vegas, certain site‐specific conditions may make DPR more attractive in other locations.

## 1 INTRODUCTION

Once considered an “option of last resort” (National Research Council [NRC], 1998), potable reuse has emerged as a viable alternative for addressing water scarcity and uncertainty throughout the world. Building on the long‐term success of benchmark systems (Gerrity, Pecson, Trussell, & Trussell, 2013), potable reuse is starting its upward trajectory on the technology diffusion “S” curve (Kiparsky, Sedlak, Thompson, & Truffer, 2013). This has been facilitated by the establishment of “pragmatic legitimacy” (perceived benefits) and “moral legitimacy” (successful track record of agency in question) in many instances, but “cognitive legitimacy” is often still lacking (Harris‐Lovett, Binz, Sedlak, Kiparsky, & Truffer, 2015). This means that the public—and even some stakeholders—will often default to other alternatives (e.g., conservation, desalination) before identifying potable reuse as a potential solution to water supply challenges (Rock, Solop, & Gerrity, 2012). Beyond public perception, the broad implementation of potable reuse can be hindered by incomplete or nonexistent regulatory frameworks, economic concerns, and/or site‐specific conditions that preclude certain treatment technologies (NRC, 2012). That being said, industry stakeholders are developing strategies to eliminate or mitigate any remaining institutional barriers to potable reuse (Meridien, 2018). Moreover, in the United States, the US Environmental Protection Agency (USEPA) recently acknowledged the important role of potable reuse in augmenting water supplies (USEPA, 2017).

When considering various water supply alternatives, the evaluation and optimization of life cycle cost can be invaluable to the decision‐making process (Bradshaw, Ashoori, Osorio, & Luthy, 2019). A more comprehensive triple bottom line analysis might even be warranted because of its ability to simultaneously consider the social, environmental, and economic implications of an engineering design (Haak, Sundaram, & Pagilla, 2018; Schimmoller, Kealy, & Foster, 2015; Schoen et al., 2017). With respect to social considerations, the recent literature demonstrates that, when designed and operated properly, potable reuse systems provide adequate protection of public health (Amoueyan, Ahmad, Eisenberg, & Gerrity, 2019; Amoueyan, Ahmad, Eisenberg, Pecson, & Gerrity, 2017; Chaudhry, Hamilton, Haas, & Nelson, 2017; Pecson et al., 2017; Pecson, Trussell, Pisarenko, & Trussell, 2015; Soller, Eftim, Warren, & Nappier, 2017). Particularly in California, potable reuse treatment trains often use both low‐pressure and high‐pressure (i.e., reverse osmosis [RO]) membranes, but when not mandated by local regulations or necessitated by salinity management, the use of membranes may lead to excessive costs or overall sustainability concerns (Bradshaw et al., 2019; Schimmoller et al., 2015). Alternative treatment trains using ozone (O_3_)‐biofiltration have been identified as “equivalent” on the basis of public health (Trussell, Salveson, Snyder, Trussell, & Gerrity, 2016) and are more cost and energy efficient (Gerrity et al., 2014; Herman, Scruggs, & Thomson, 2017), but these benefits must be evaluated against certain water quality limitations, including higher concentrations of total organic carbon (TOC) and total dissolved solids (TDS) in the final product water. Conversely, RO will achieve low concentrations of TOC and TDS but will likely lead to greater energy consumption, greenhouse gas (GHG) emissions, and environmental discharge of concentrated brine streams.

In addition to discussions of treatment trains, stakeholders must determine whether indirect potable reuse (IPR) or direct potable reuse (DPR) is more appropriate for a particular system (Herman et al., 2017). In the case of IPR, which is historically the more common approach (Gerrity et al., 2013), water agencies must often evaluate the pros and cons of groundwater replenishment (via spreading or direct injection) against those of surface water augmentation. In the United States, large‐scale DPR has only been implemented in Texas (Gerrity et al., 2013), but other states, including California (State Water Resources Control Board [SWRCB], 2018), Arizona (National Water Research Institute [NWRI], 2018), and New Mexico (NWRI, 2016), are in the process of developing their own DPR regulations, which will eventually allow even greater flexibility in the water supply decision‐making process. In the city of San Diego, for example, a DPR approach would eliminate the pipeline and pumps required to lift the purified water to a local reservoir (formerly San Vicente Reservoir but now Miramar Reservoir) as proposed in the IPR configuration. Based on the original San Vicente Reservoir design, DPR could reduce capital costs by as much as US$100 million and operational costs by as much as $1,250/acre‐foot (AF) (Raucher & Tchobanoglous, 2014). Despite the additional costs, IPR is still justifiable considering its greater cognitive legitimacy relative to DPR and the prediction that conventional imported water supplies are expected to reach $2,000/AF in the near future (Raucher & Tchobanoglous, 2014). Therefore, the decision to pursue potable reuse in its many forms requires consideration of numerous site‐specific factors that span society, economy, and the environment (NRC, 2012).

**Figure 1:**
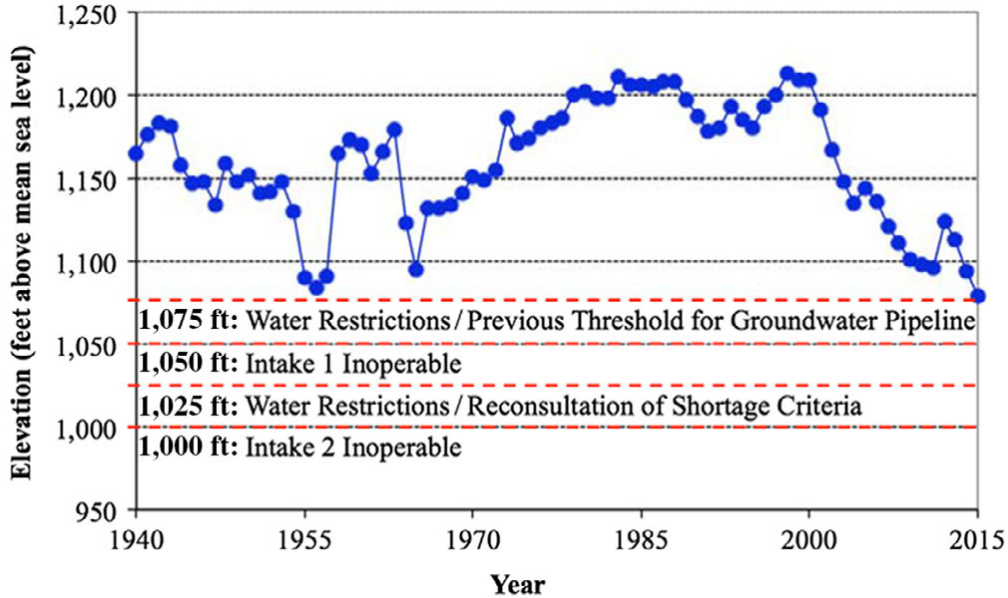
Historical elevation of Lake Mead at Hoover Dam with notable elevation thresholds. A constant elevation of 1,075 ft (Nevada Colorado River allocation = 300,000 acre‐feet [AF]) was assumed for the baseline scenarios. For the “shortage” scenario, the elevation was assumed to decrease linearly from 1,075 to 1,050 ft (Nevada Colorado River allocation = 287,000 AF) from 2015 to 2025 and then remained constant until 2065. An elevation of 1,050 ft was previously identified as the “deadpool” elevation for hydroelectric power generation, but that has been revised down to 950 ft after the installation of more efficient turbines. A recently constructed third intake allows for local withdrawals down to an elevation of 860 ft, although discharges via the Hoover Dam are no longer possible below 895 ft

**Figure 2:**
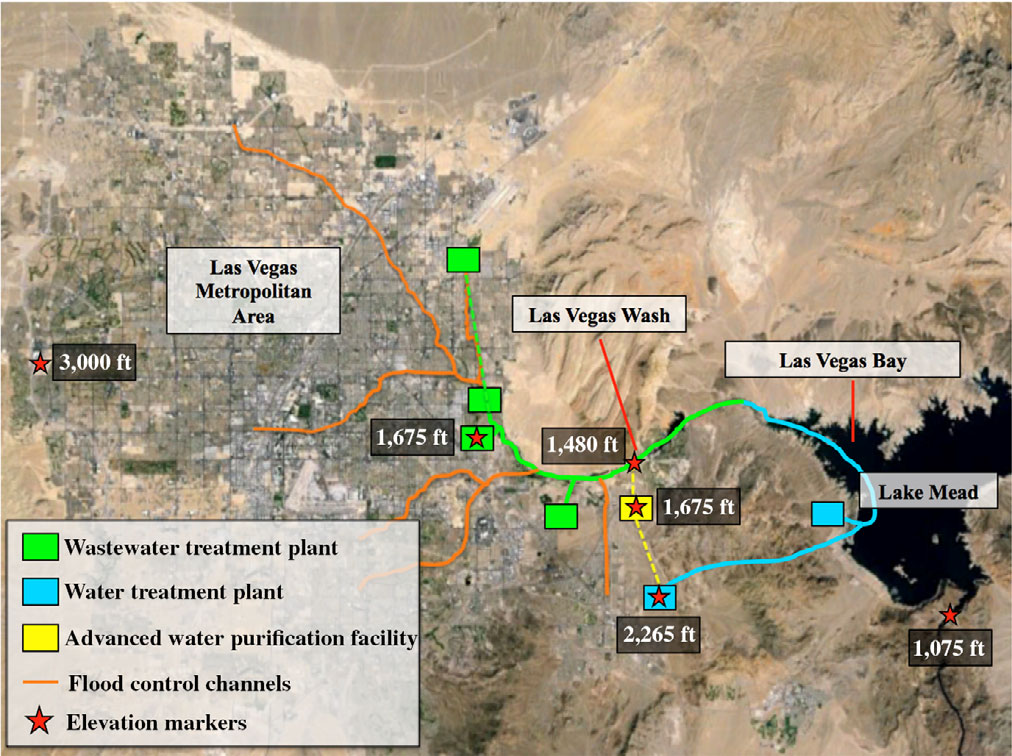
Map of the Las Vegas metropolitan area and relevant water, wastewater, and storm water infrastructure and associated elevations. Solid lines represent surface water flow, and dashed lines represent pipelines. The advanced water purification facility and associated pipeline represent hypothetical infrastructure for implementation of direct potable reuse. *Source*: Google Earth Pro and DigitalGlobe

The Las Vegas metropolitan area offers an interesting case study for evaluating the sustainability of water supply alternatives (Stave, 2003; Venkatesan, Ahmad, Johnson, & Batista, 2011a). For the past several decades, the southwestern United States has experienced rapid population growth and record drought, with Lake Mead recently dropping to its lowest water elevation in history (Bureau of Reclamation, 2018) (Figure 1). Las Vegas is almost entirely dependent on Lake Mead for its municipal water supply, with an annual consumptive allocation of 300,000 AF. The Southern Nevada Water Authority (SNWA) maximizes this allocation through the use of “return flow credits” (RFCs), which allow matching withdrawals for treated wastewater returned to Lake Mead via the natural drainage path known as the Las Vegas Wash (Figure 2). However, the difference in elevation between Lake Mead and the Las Vegas valley (>1,190 ft assuming a lake elevation of 1,075 ft) translates into a considerable energy expenditure just to recapture the RFCs as drinking water. With respect to capital improvements, SNWA recently constructed a third intake and is currently expanding the associated pump station at a total cost of ~$1.5 billion to ensure access to the lake even if the elevation drops below 1,000 ft (Brean, 2017). Moreover, as the lake initially approached an elevation of 1,075 ft, SNWA considered the construction of a 500‐km pipeline at an estimated construction cost of ~$3.2 billion to transfer groundwater from central Nevada to Las Vegas (i.e., the Nevada Groundwater Development Project; SNWA, 2011).

The uncertain hydrological conditions of the Colorado River system offer pragmatic legitimacy to other water supply alternatives, including the aforementioned capital improvement projects and potential future implementation of DPR. However, it is currently unclear whether DPR could be implemented at a lower cost than the status quo IPR/RFC approach while still ensuring adequate supply and drinking water quality. Nevada recently revised its regulations to permit IPR via groundwater replenishment (the RFC approach was specifically excluded to ensure that this historical practice was not impacted) (Nevada Division of Environmental Protection [NDEP], 2016), but DPR has not yet been regulated in Nevada. Nevertheless, with recent momentum in surrounding states, DPR could eventually become a reality in Nevada if a need was demonstrated. As such, the goal of this case study was to present a framework for evaluating the sustainability of IPR versus DPR (with alternative treatment trains) and to determine whether DPR is a viable option for Las Vegas considering its site‐specific factors. The viability of each alternative was evaluated based on its ability to (1) meet projected water demands, (2) maintain or improve drinking water quality, (3) reduce energy consumption and overall costs, and (4) reduce potential environmental impacts associated with wastewater discharges.

## 2 METHODOLOGY

### 2.1 Las Vegas metropolitan area water system and modeling approach

**Figure 3:**
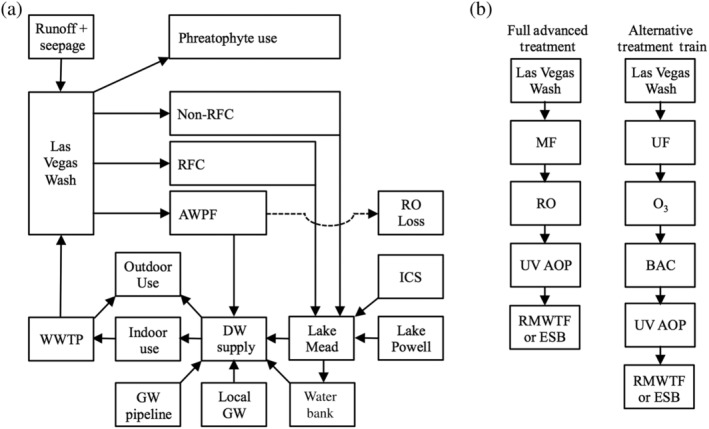
(a) Simplified schematic of the system dynamics model describing water supply and water quality in the Las Vegas metropolitan area. (b) Treatment trains considered for the advanced water purification facility (AWPF) in the direct potable reuse (DPR) scenarios. For DPR with raw water augmentation, the purified water from the AWPF was pumped to the River Mountains Water Treatment Facility (RMWTF) for additional treatment prior to distribution. For DPR with finished water augmentation, the purified water from the AWPF was sent to an engineered storage buffer (ESB) prior to direct distribution. BAC, biological activated carbon; DW, drinking water; ICS, intentionally created surplus; GW, groundwater; MF, microfiltration; O_3_, ozone; RFC, return flow credits; RO, reverse osmosis; UF, ultrafiltration; UV AOP, ultraviolet advanced oxidation process; WWTP, wastewater treatment plant

The water system for the Las Vegas metropolitan area is summarized in Figure 2 and described in greater detail in the following sections. This information was incorporated into a stock‐and‐flow system dynamics model using the Stella 10.1 software package (ISEE Systems, Lebanon, NH). The system dynamics model simulated population growth; water quantity (supply and demand); water quality (TDS and total phosphorus [TP]); and energy consumption, energy costs, and GHG emissions. A simplified model schematic is provided in Figure 3a, and more detailed stock and flow diagrams are provided in Figures S2–S6. The target study period was 2015–2065 (delta time interval of 0.25 years in Stella), but the model also included the preceding 10‐year period to ensure model stabilization and output consistent with actual conditions observed in 2015. Further validation was achieved by comparing model output for the baseline scenario against independent literature and, more specifically, by recreating conditions observed and forecasted for Las Vegas (SNWA, 2015; Stave, 2003).

### 2.2 Baseline water supply

Under normal conditions, Nevada has a Colorado River consumptive use allocation of 300,000 acre‐feet per year (AFY), although this number can decrease depending on the elevation of Lake Mead. The allocation initially decreases to 287,000 AFY if the elevation of Lake Mead drops below 1,075 ft (Bureau of Reclamation, 2007a). For the baseline scenario, the water elevation in Lake Mead was assumed to remain constant at 1,075 ft for the duration of the study period to eliminate the need for complex hydrological considerations. An initial volume of 9.6 million AF (MAF), consistent with the 1,075 elevation, was also assumed for Lake Mead.

Water withdrawals from Lake Mead, which currently comprise 90% of the total water supply, are primarily treated at the Alfred Merritt Smith Water Treatment Facility (AMSWTF) or the River Mountains Water Treatment Facility (RMWTF) using ozonation, ferric chloride coagulation, flocculation, chlorination, and dual media filtration. The combined design capacity is approximately 1 MAF/year, and treatment costs are approximately $4/AF for energy and $5/AF for chemicals (Cooley & Wilkinson, 2012; Text S1). Treated surface water is supplemented with approximately 46,830 AFY of local groundwater (SNWA, 2015). Groundwater treatment costs were assumed to be negligible for this study (Cooley & Wilkinson, 2012).

### 2.3 Population and water demand projections for baseline scenario

**Figure 4:**
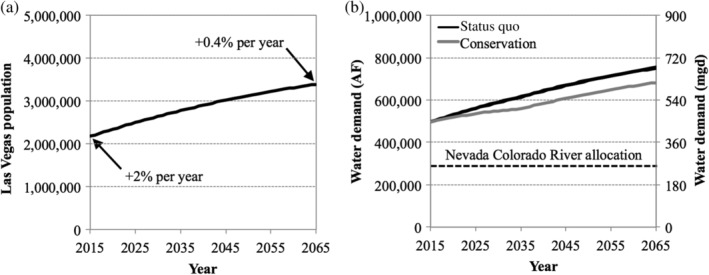
(a) Population projections for Las Vegas based on Equation (S1) and (b) the corresponding water demand based on estimated per capita water use under the status quo and conservation scenarios

Long‐term population projections (2015–2050) for Clark County (Tra, 2015) were used to develop a polynomial regression (Equation (S1)) to project the population through 2065 (Figure 4a). The corresponding water demand was calculated based on current per capita water use and future projections based on published conservation targets. As of 2014, total water use was approximately 205 gpcd, but SNWA is aiming to reduce this value to 199 gpcd by 2035 (SNWA, 2015). Starting in 2015, the model assumed a linear reduction in per capita water use until reaching the 2035 target, at which point the per capita water use was held constant at 199 gpcd. Coupled with the population projections, this resulted in 2015 and 2065 water demands of approximately 500,000 and 750,000 AFY, respectively (Figure 4b). For the status quo or baseline condition, it was assumed that 56% of the water supply was used outdoors (i.e., consumptive use), and 44% was used indoors (i.e., collected and treated at a local wastewater treatment plant [WWTP]) (SNWA, 2015).

### 2.4 Wastewater treatment and return flow credits

It was assumed that all water used indoors was captured and treated at one of four major WWTPs (Figure 2). These WWTPs all use activated sludge processes, media and/or membrane filtration, and disinfection prior to discharge to the Las Vegas Wash or to sites irrigating with recycled water (e.g., golf courses). There are also several satellite water reclamation facilities that produce recycled water for municipal, commercial, and industrial nonpotable reuse (not shown in Figure 2). A constant 21,800 AFY of treated wastewater effluent was allocated to consumptive nonpotable reuse in the Stella model for the duration of the study period (SNWA, 2015). The remaining treated wastewater flows were allocated to the Las Vegas Wash, along with a constant flow of urban runoff and groundwater seepage of 22,193 AFY (SNWA, 2015).

As mentioned earlier, southern Nevada currently leverages the RFC concept to extend its Colorado River water supply—a form of IPR. In the Stella model, the RFC total included all flows in the Las Vegas Wash with the exception of urban runoff and groundwater seepage (22,193 AFY), which are represented as “non‐RFCs” in Figure 3a. A base flow of 12,000 AFY was also maintained in the Las Vegas Wash at all times—even for the 100% DPR scenarios—for phreatophyte use (Figure 3a). This represents a reasonable approximation of the current RFC calculation methodology (Bureau of Reclamation, 2007b). The previous calculation methodology excluded flows originating outside the Colorado River system (e.g., local groundwater; Bureau of Reclamation, 1984) but was revised in 2007 to account for future importation of surface water and groundwater into Las Vegas.

### 2.5 Direct potable reuse

DPR can be implemented in a wide range of configurations, including “flange‐to‐flange,” blending advanced treated wastewater effluent (hereafter *purified water*) immediately upstream of a drinking water treatment plant (i.e., raw water augmentation), or blending purified water with finished drinking water (i.e., finished water augmentation) (SWRCB, 2018). Typically, DPR eliminates the discharge of purified water to an environmental buffer. However, a system may also be considered DPR if the environmental buffer fails to achieve the storage times and/or dilution ratios required in an IPR configuration (SWRCB, 2016).

In the configuration proposed for this study, conventionally treated wastewater effluent was discharged to the Las Vegas Wash, and varying percentages of the Las Vegas Wash flow (25%, 50%, 75%, and 100%) were pumped in a hypothetical pipeline to an advanced water purification facility (AWPF). For reference, this configuration would not satisfy California's minimum storage times (>60–180 days) or maximum recycled water contributions (<1%–10%) required for IPR via surface water augmentation (California Division of Drinking Water [California Division of Drinking Water], 2017), thereby leading to a DPR designation.

Two different treatment trains were considered for the AWPF in the DPR scenarios (Figure 3b). The first treatment train (DPR 1) included microfiltration (MF), RO, and an ultraviolet advanced oxidation process (UV AOP)—a combination that satisfies California's “full advanced treatment” requirement (DDW, 2017). The RO process was assumed to achieve 90% water recovery and 99% TDS rejection (DDW, 2017). Overall recovery from conventional MF‐RO might be as low as 70%–80% (Bradshaw et al., 2019; Trussell et al., 2016), but 90% was assumed to be a best‐case scenario. The second treatment train (DPR 2), which is a potentially more sustainable alternative (Gerrity et al., 2014), included ultrafiltration (UF), O_3_, biological activated carbon (BAC), and UV AOP. The O_3_ dose was assumed to be approximately 9 mg/L to achieve an O_3_/TOC ratio of 1.0 (Gerrity et al., 2014), which likely represents a conservative estimate from a cost perspective. Specifying other operational or dosing parameters was unnecessary for the purposes of this study. Coupled with the conventional WWTPs, each of these DPR treatment trains is capable of achieving the pathogen log removal values recommended for potable reuse, even without the additional treatment at RMWTF (Amoueyan et al., 2019; Soller et al., 2017; Tchobanoglous et al., 2015), but there are other water quality implications that will be discussed later.

In the raw water augmentation scenario, the purified water from the AWPF was pumped to the RMWTF in a hypothetical 9‐km‐long pipeline and blended with raw surface water prior to drinking water treatment (Figure 2). For the finished water augmentation alternative, it was assumed that purified water from the AWPF was sent to an engineered storage buffer (ESB) with an 8‐h response retention time prior to direct distribution. The blending ratio was assumed to be the same as the RMWTF configuration. It was assumed that this eliminated 75% of the capital and operational costs associated with the DPR pipeline and the additional treatment costs at the RMWTF, thereby resulting in a lower net present worth (NPW) alternative.

### 2.6 Intentionally created surplus

Through various intrastate, interstate, and international agreements, SNWA has acquired intentionally created surplus (ICS) water rights that can be developed over time to supplement its water supply (SNWA, 2015). As of 2015, SNWA had acquired 27,200 AFY of tributary conservation ICS (i.e., in‐state tributaries to the Colorado River) and 9,000 AFY of imported ICS (i.e., groundwater that can be redirected to the Colorado River), both of which are eligible for RFCs and can be accessed during declared shortages. SNWA is also eligible for system efficiency ICS, extraordinary conservation ICS, and binational ICS, but consistent with SNWA's water resource plan (SNWA, 2015), these resources were not considered in the model due to various restrictions on use and expiration dates. Therefore, the model considered only tributary conservation ICS and imported ICS, which were initiated once the demand calculated by the model exceeded the baseline supply (i.e., Nevada's Colorado River allocation, RFCs, local groundwater, and DPR when applicable).

### 2.7 Water banks (temporary resources)

During times of need, SNWA can also access temporary resources that have been “banked” locally or through agreements with Arizona and California. A total of 1,267,000 AF had been banked as of 2016 (SNWA, 2017a). During periods of withdrawal, Arizona and California would forego their Colorado River allocation, thereby allowing Nevada to withdraw additional water from Lake Mead at a maximum rate of up to 90,000 AFY. With respect to the model, any surplus supply from Lake Mead was diverted to the bank (starting in 2015), and withdrawals were initiated as needed (also starting in 2015) once demand exceeded the baseline supply plus ICS. These resources were also eligible for RFCs.

### 2.8 Water quality considerations

Because Lake Mead has been identified as a phosphorus‐limited reservoir (Ding, Hannoun, List, & Tietjen, 2014), the local WWTPs and other nonpoint sources are limited to total maximum daily loads (TMDLs) of 151 and 45 kg/day of TP, respectively, for the months of March through October (NDEP, 2003). For the model, both loadings were held constant at these TMDLs throughout the year (consistent with actual WWTP operations), which ultimately required reductions in wastewater effluent TP concentration as the wastewater flows increased over time (Figure S6). For the DPR scenarios, the WWTPs still maintained compliance with the TMDL, even though the treated wastewater may have ultimately been diverted to the AWPF.

Because a corresponding hydrodynamic/hydrological model for Lake Mead was beyond the scope of the current study, TDS was not modeled with a closed‐loop approach. The initial condition for the TDS concentration in Lake Mead was selected to target a concentration of 600 mg/L in 2015 (SNWA, 2017b), and the TDS concentration in Lake Mead was assumed to increase at a constant annual rate of 0.25% for the duration of the study period (Colorado River Basin Salinity Control Forum, 2017). TDS inputs to wastewater originated from human waste (52 kg/person‐year) and water softeners (80 kg/softener‐year), with 30% of the population assumed to use a water softener (Venkatesan, Ahmad, Johnson, & Batista, 2011b). In addition to inputs from wastewater effluent discharge, urban runoff and seepage were assumed to contribute 192,000 metric tons per year to the Las Vegas Wash (Venkatesan et al., 2011b).

The TDS concentration of the finished drinking water was affected by the annual increase of 0.25% in Lake Mead; TDS loadings from groundwater supplies (local = 300 mg/L [Dettinger, 1987] and pipeline = 389 mg/L [Schaefer, Thiros, & Rosen, 2006]); and inputs from any DPR flows, which initially received water from the Las Vegas Wash. The RO‐based treatment train was assumed to achieve 90% water recovery and 99% TDS rejection, while the non‐RO treatment train was assumed to achieve no reduction in TDS relative to the initial concentration in the Las Vegas Wash.

### 2.9 Energy consumption and GHG emissions

The RMWTF, which is located at an elevation of 2,265 ft, was selected as the reference point for energy calculations in the current study (Figure 2). Because this study focused primarily on the benefits of RFC diversion, calculations for the RFC models only considered the energy consumption associated with pumping (Equations (S2) and (S3)) and treating RFCs at the RMWTF (Text S1). For the DPR models, calculations considered energy consumption associated with pumping and treating RFCs at the RMWTF (when applicable), advanced treatment at the AWPF, pumping purified water in the DPR pipeline, and further treatment of the purified water at the RMWTF (when applicable). The elevation of the Las Vegas Wash diversion point was assumed to be 1,480 ft to maximize potential DPR withdrawals, and the elevation of the AWPF was assumed to be 1,675 ft (Figure 2). Energy costs for the treatment of other surface water withdrawals (e.g., baseline Colorado River allocation) were assumed to be the same for all scenarios, so they were not considered in the model. Energy consumption for the AWPF was based on literature values and full‐scale data from existing facilities in California (Table S1): MF/UF = 295 kWh/AF, RO = 504 kWh/AF, UV AOP = 85 kWh/AF, O_3_ = 128 kWh/AF, and BAC = 31 kWh/AF (Gerrity et al., 2014; Raucher & Tchobanoglous, 2014). Based on Nevada's 2015 energy portfolio, which primarily relies on natural gas, electricity generation resulted in a carbon intensity of 0.38 kg CO_2e_ per kWh (Text S1; U.S. Energy Information Administration, 2017). This carbon intensity will likely decrease over time, but due to uncertainty in these changes, the value was assumed to be constant over the study period. For monetization of GHG emissions, the mid‐case CO_2e_ price trajectory in Luckow et al. (2015) was linearized to establish CO_2e_ price estimates ranging from $7/metric ton in 2015 to $129/metric ton in 2065.

### 2.10 Capital and operations and maintenance costs

Capital costs were estimated for each DPR treatment train, the DPR pipeline to the AWPF and RMWTF, and the Nevada Groundwater Development Project (i.e., groundwater pipeline; described later). Capital costs for the DPR treatment trains were developed using the conceptual‐level class 4 (Association for the Advancement of Cost Engineering, 2011) approach from Plumlee, Stanford, Debroux, Hopkins, and Snyder (2014), which provides an accuracy of −30% to +50%. The unit cost equations for the DPR treatment processes are summarized in Table S2, and the cost estimation approach for the DPR pipeline is summarized in Tables S4 and S5. For each DPR scenario, the AWPF was constructed in two phases based on model output for the DPR flow rate: (1) initial construction in 2015 for the 2035 design flow and (2) an expansion in 2035 for the 2065 design flow. The DPR pipeline was constructed in 2015 for the 2065 design flow, and the pipeline diameter was selected to limit water velocity to a maximum of 2 m/s. For finished water augmentation (i.e., direct distribution), an additional $1.25/gal was added for an engineered storage buffer (ESB capacity for each scenario shown in Table S6) assuming an 8‐h response retention time (Tchobanoglous et al., 2015).

As mentioned earlier, costs for conventional drinking water treatment at the AMSWTF and RMWTF were assumed to be $9/AF (Text S1), and pumping costs for all scenarios were calculated based on model output and SNWA's historical electricity cost of $0.05/kWh (Giltner, 2019). For the AWPF, overall annual operations and maintenance (O&M) costs, including electricity, replacement parts, chemicals, and labor, were determined using modified unit cost curves from Plumlee et al. (2014) (Table S3). The modified curves were developed from the raw data in Plumlee et al. (2014) assuming an electricity cost of $0.05/kWh instead of $0.0988/kWh. For RO, an additional $155/AF was included for brine disposal via evaporation ponds (Raucher & Tchobanoglous, 2014).

The unit cost curves in Plumlee et al. (2014) were actually based on 2011 U.S. dollars. The estimated costs were first adjusted to 2015 U.S. dollars using the relevant construction cost index (ENR, 2018), and further adjustments for the time value of money were based on a discount rate of 3% for consistency with Schimmoller et al. (2015). Similar adjustments were made for the Nevada Groundwater Development Project and the DPR pipeline. Additional details are provided in Text S1.

### 2.11 Supplementary modeling scenarios

In addition to the baseline (status quo) RFC approach and the hypothetical DPR scenarios, the model was modified to evaluate (1) the proposed Nevada Groundwater Development Project (i.e., groundwater pipeline scenario), (2) a further decline in water elevation in Lake Mead (i.e., shortage scenario), and (3) a conservation scenario.

#### 2.11.1 Groundwater pipeline scenario

As mentioned earlier, SNWA previously considered the construction of a 500‐km pipeline to transfer groundwater from central Nevada to Las Vegas at an estimated cost of $3.2 billion in 2007 U.S. dollars. Construction of this pipeline was originally expected to begin once the elevation of Lake Mead dropped below 1,075 ft, but the project has since been suspended. With respect to the model, construction and implementation of the pipeline was initiated once demand exceeded the baseline supply plus ICS but before withdrawing banked resources. The pipeline was assumed to provide up to 134,434 AFY (SNWA, 2011) and was also eligible for RFCs (SNWA, 2015). DPR was not considered in this scenario because the combined costs were assumed to be prohibitive. Only the capital costs for construction of the pipeline and the costs associated with pumping and treating the resulting RFCs were considered in the groundwater pipeline scenario. Comparable to the Hetch Hetchy system in California, for which energy costs are estimated at $0.03/AF (Cooley & Wilkinson, 2012), the Nevada Groundwater Development Project will rely largely on gravity flow, thereby resulting in negligible O&M costs.

#### 2.11.2 Lake Mead shortage scenario

The baseline scenarios assumed a constant Lake Mead elevation of 1,075 ft. The shortage scenario assumed a constant elevation of 1,075 ft until 2015, followed by a linear decrease over 10 years to 1,050 ft and then a constant elevation of 1,050 ft until 2065. This was implemented for the baseline RFC scenario and for the DPR 1 scenario with 100% diversion to the AWPF. This allowed for an evaluation of water supply effects and impacts on RFC pumping costs.

#### 2.11.3 Conservation scenario

As discussed later, the baseline RFC scenario resulted in water bank depletion and an overall water supply deficit at the end of the study period. The conservation scenario was used to identify the required reduction in outdoor water consumption that would allow all demands to be satisfied without the use of temporary resources from the water bank. In the baseline scenarios, per capita demands decreased linearly from 205 gpcd in 2015 to 199 gpcd in 2035, with outdoor water use accounting for a constant 56% of overall water use. In the conservation scenario, per capita demand decreased linearly from 205 gpcd in 2015 to 181 gpcd in 2035 (Figure 4b), which was accomplished with a 21% reduction in outdoor water use, while indoor water use remained constant at 90 gpcd for the entire study period. The model automatically adjusted the outdoor water use ratio (from a maximum of 56% to a minimum of 50%) to match the change in use pattern. Realistically, there would be concurrent reductions in indoor water use, but due to the limited impact of indoor conservation on the Las Vegas water supply (Stave, 2003), the conservation scenario focused only on outdoor conservation efforts.

## 3 RESULTS AND DISCUSSION

### 3.1 Water supply

**Figure 5:**
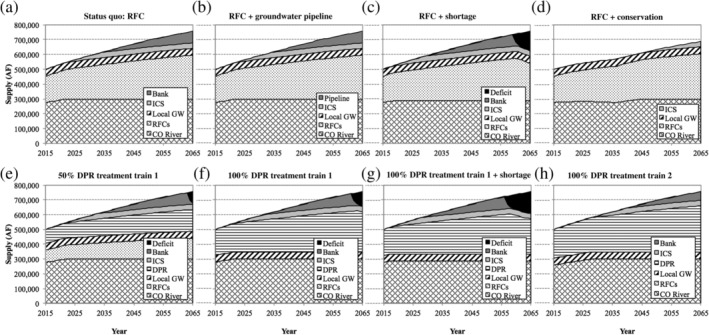
Composition of the Las Vegas water supply for (a–d) return flow credits‐only scenarios and (e–h) direct potable reuse scenarios. The water supply deficit of 0.019 million acre‐foot for the status quo RFC scenario (see Table 1) is too small to be visible in (a). CO River, Colorado River; DPR, direct potable reuse; GW, groundwater; ICS, intentionally created surplus; RFC, return flow credit

The model was first used to evaluate the ability of the southern Nevada water system to meet projected demands using permanent, temporary, and future/hypothetical water supplies. Figure 5 summarizes the results from a subset of the modeling scenarios. For the status quo RFC approach (Figure 5a), demand exceeded the permanent supply starting in 2035, thereby requiring withdrawals from the water bank. These temporary resources extended the water supply out to 2064, but there was a net deficit of 19,000 AF as of 2065. These results are consistent with SNWA (2015).

Consistent with the status quo RFC scenario, the model initiated the pipeline for the Nevada Groundwater Development Project in 2035 (Figure 5b), and the imported groundwater flows sufficiently augmented the overall water supply to meet demands for the entire study period. The groundwater importation rate reached a maximum of 77,000 AFY in 2065, which is considerably lower than the maximum allocation of 134,434 AFY.

In the shortage scenario (Figure 5c), there was an immediate decrease in the Colorado River allocation to 287,000 AFY, which necessitated the use of temporary resources 5 years earlier in 2030. Demand exceeded the overall water supply starting in 2059, thereby resulting in a total deficit for the study period of 0.6 MAF.

Finally, by reducing outdoor water use by 21% in the conservation scenario (Figure 5d), demand was satisfied for the entire study period. ICS use was delayed until 2046, but because ICS withdrawals reached their maximum allocation in 2065, bank withdrawals would be initiated starting in 2066. Because of the reduced water demand during the study period, an additional 0.5 MAF were banked between 2015 and 2046 to supplement the initial 1.3 MAF. This is indicated by the dip in Colorado River withdrawals in Figure 5d.

DPR's contribution to the overall water supply increased for higher diversion percentages, with corresponding reductions in the RFC contribution (Figure 5e,f). The DPR product water flow rates ranged from 66 to 281 million gallons per day (mgd) at buildout (Figure S1). Similar to the status quo RFC approach, DPR 1 was unable to satisfy demand toward the end of the study period, and the overall deficits ranged from 0.1 MAF for the 25% diversion to 0.3 MAF for the 100% diversion. Similarly, DPR 1 under the shortage condition resulted in a net deficit of 1.0 MAF (Figure 5g) compared with the 0.6 MAF deficit for the corresponding RFC scenario. The RO process in DPR 1 diverted 10% of the feed water to evaporation ponds, but this was mitigated to some degree by the capture of non‐RFCs, specifically the urban runoff and seepage that were excluded from the RFC water supply to simulate the Bureau of Reclamation accounting procedure. Because there was no water loss with DPR 2 (i.e., no RO), the additional capture of non‐RFCs allowed DPR 2 to meet projected demands for all scenarios (e.g., Figure 5h). All deficits are summarized in Table 1.

**Table 1:** Summary of water supply deficits and net present worth (NPW) for each modeling scenario

Scenario	Total deficit (MAF)	Phase 1 capital (US$M)	Phase 2 capital (US$M)	NPW (US$M)	NPW (US$/AF of RFC + DPR)	NPW (US$/AF of DPR)
RFC	0.019	N/A	N/A	$559	N/A	$45
RFC + pipeline (in 2035)	0.000	$2,215	N/A	$2,774	N/A	$225
RFC + shortage	0.618	N/A	N/A	$565	N/A	$46
RFC + conservation	0.000	N/A	N/A	$574	N/A	$45
DPR with raw water augmentation						
DPR 1 25%	0.085	$314	$51	$1,636	$391	$134
DPR 1 50%	0.167	$534	$87	$2,503	$358	$206
DPR 1 75%	0.255	$734	$117	$3,310	$340	$275
DPR 1 100%	0.329	$884	$145	$4,042	$338	$338
DPR 1 100% shortage	1.036	$877	$126	$3,866	$328	$328
DPR 2 25%	0.000	$223	$35	$1,071	$190	$85
DPR 2 50%	0.000	$379	$60	$1,446	$170	$113
DPR 2 75%	0.000	$522	$82	$1,789	$160	$137
DPR 2 100%	0.000	$630	$103	$2,054	$155	$155
DPR with finished water augmentation^a^						
DPR 1 25%	0.085	$313	$54	$1,569	$370	$128
DPR 1 50%	0.167	$546	$92	$2,382	$338	$196
DPR 1 75%	0.255	$758	$126	$3,135	$322	$260
DPR 1 100%	0.329	$921	$156	$3,827	$320	$320
DPR 1 100% shortage	1.036	$919	$136	$3,655	$310	$310
DPR 2 25%	0.000	$224	$38	$1,000	$169	$79
DPR 2 50%	0.000	$395	$66	$1,316	$151	$102
DPR 2 75%	0.000	$553	$92	$1,599	$142	$122
DPR 2 100%	0.000	$675	$116	$1,821	$137	$137

*Note*: All costs are present worth estimates in 2015 U.S. dollars assuming a discount rate of 3% and a study period spanning 2015–2065.

Abbreviations: AF, acre‐foot; DPR, direct potable reuse; DPR 1, DPR treatment train 1; DPR 2, DPR treatment train 2; MAF, million acre‐foot; RFC, return flow credit.

a Capital costs are higher because of the engineered storage buffer.

The model demonstrated that temporary resources should be sufficient to extend the southern Nevada water supply out to at least 2059, even during a shortage condition, but the demand associated with the growing population would eventually deplete the water bank unless additional water policies were implemented. According to Gerrity and Snyder (2011), the Las Vegas metropolitan area generates approximately $460,000 in gross metropolitan product (in 2015 U.S. dollars) for every million gallons of water withdrawals. The shortages in Table 1 would amount to a minimum net present loss of $650 million for the baseline RFC scenario and $21 billion for the shortage scenario. Strictly from a supply perspective, the proposed groundwater pipeline, reductions in outdoor water use, and/or DPR (with non‐RO treatment trains) could be implemented to further extend the water supply, reduce reliance on temporary resources, and mitigate potential economic consequences. However, there are other issues, including water quality, energy consumption, and economics, that might preclude these alternatives from consideration.

### 3.2 Water quality

One of the major water quality concerns in the southwestern United States is the high TDS concentration of many drinking water supplies, which stems from a combination of evaporation and highly saline discharges into the Colorado River as it travels to Mexico. In fact, Las Vegas drinking water already exceeds the USEPA's secondary standard of 500 mg/L for TDS. This can be contrasted with water supplies sourced primarily from nearby snowmelt, such as in Reno, Nevada, where even the treated wastewater effluent sometimes contains less than 400 mg/L of TDS (Mortensen, Cath, Brant, Dennett, & Childress, 2007).

As shown in Figure S7, the TDS concentration in Lake Mead was modeled with a constant annual increase of 0.25%, which resulted in concentrations of 600 mg/L in 2015 and 680 mg/L in 2065. Due to a small amount of dilution from local groundwater, the drinking water concentration for the status quo RFC scenario ranged from 570 mg/L in 2015 to 650 mg/L in 2065. The more problematic issue from a DPR perspective was the considerable increase in TDS in the wastewater effluent and, ultimately, the Las Vegas Wash due to salt loads from human inputs, water softeners, and runoff/seepage. This led to TDS concentrations in Las Vegas Wash—the feed water for DPR—ranging from 1,660 to 1,760 mg/L during the study period.

**Figure 6:**
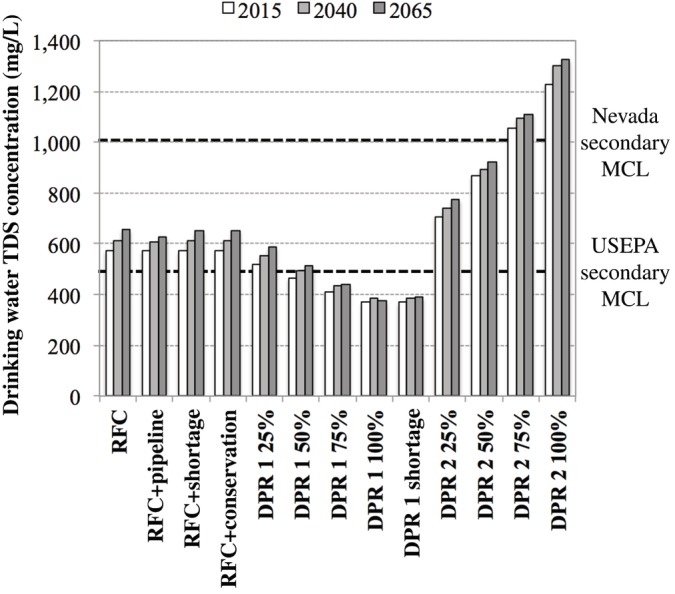
Total dissolved solids concentration of the blended drinking water for each scenario. DPR, direct potable reuse; DPR 1, DPR treatment train 1; DPR 2, DPR treatment train 2; MCL, maximum contaminant level; RFC, return flow credit; TDS, total dissolved solids

Although this was not a significant issue for DPR 1 using RO, DPR 2 was unable to remove any TDS from the Las Vegas Wash feed water. This is apparent in Figure 6, which illustrates the change in TDS concentration for the blended drinking water supply in each scenario. All of the RFC‐only scenarios exhibited similar TDS profiles, which increased from 570 to 650 mg/L. Due to the 99% salt rejection achieved by RO, DPR 1 with a 100% diversion achieved TDS concentrations lower than the USEPA secondary standard of 500 mg/L. On the other hand, the blended drinking water for the DPR 2 scenario contained TDS concentrations approaching 1,400 mg/L with 100% diversion.

Therefore, DPR 2 with higher diversion percentages was able to satisfy demand throughout the study period, but the water supply benefits were clearly offset by the elevated TDS concentration, which makes this option infeasible in locations with high‐TDS source waters (e.g., Las Vegas). The TDS issue would also be compounded by other water quality concerns. Pathogens could be attenuated by optimizing upstream wastewater treatment and/or the disinfection processes at the AWPF (e.g., O_3_, UV, and free chlorine disinfection in the ESB). However, the required O_3_ dose would likely exceed an O_3_/TOC ratio of 1.0 (Gerrity et al., 2014), and this dosing level has been shown to form up to 30 μg/L of bromate in local wastewater (Lee et al., 2016). Considering that the DPR flow accounted for up to 10%–40% of the overall water supply for the 25% and 100% diversions, respectively, the DPR flow might add 3–12 μg/L of bromate to local drinking water. This would either exceed the USEPA maximum contaminant level (MCL) of 10 μg/L outright or when combined with the baseline level of bromate formed during ozonation at AMSWTF and RMWTF. Alternatively, bromate formation could be mitigated with the chlorine–ammonia process (Neemann, Hulsey, Rexing, & Wert, 2004) or by hydrogen peroxide addition (von Gunten, 2003), but the formation of alternative disinfection products might be a concern, the reduction in bromate might still be inadequate (Lee et al., 2016), disinfection efficacy might be compromised (Gamage, Gerrity, Pisarenko, Wert, & Snyder, 2013), and O&M costs would increase (Plumlee et al., 2014).

Although nitrosamine formation has been an issue in some potable reuse systems using ozonation (Gerrity et al., 2015), it is unlikely that nitrosamine formation would be a significant concern for the Las Vegas AWPF, particularly when considering the expected reduction during BAC and UV photolysis. However, the purified water for DPR 2 would still contain low concentrations of recalcitrant trace organic compounds (TOrCs) (Gerrity et al., 2011; Reungoat et al., 2012; Trussell et al., 2016), which could be problematic in the context of public perception. That being said, the current RFC scenario already results in the detection of some TOrCs in Lake Mead (Benotti, Stanford, & Snyder, 2010), which is consistent with other de facto reuse systems (Benotti et al., 2009; Nguyen et al., 2018). Even more problematic would be the formation of regulated disinfection byproducts, particularly total trihalomethanes (TTHMs), during free chlorine disinfection at the AWPF or after blending at RMWTF. The purified water from DPR 2 would likely have a TOC concentration greater than 4 mg/L, which might lead to exceedances of the TTHM MCL after chlorination (Arnold et al., 2018). For reference, Las Vegas drinking water typically has a TOC concentration of approximately 2.5 mg/L. It is unlikely that any of these issues would be a concern for DPR 1. In fact, DPR 1 would reduce TOrC loadings to Las Vegas Wash and Lake Mead.

### 3.3 Annual energy consumption

**Figure 7:**
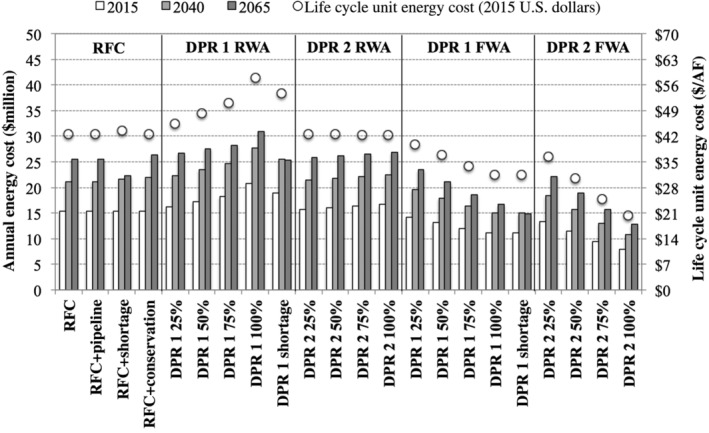
Comparison of annual energy costs and life cycle unit energy costs (standardized to the total volume of water [RFC + DPR]) across the study period. Raw water augmentation (RWA) indicates that the purified water was pumped to the River Mountains Water Treatment facility, and finished water augmentation (FWA) indicates that the purified water was pumped directly into the distribution system. DPR, direct potable reuse; DPR 1, DPR treatment train 1; DPR 2, DPR treatment train 2; RFC, return flow credit

With respect to pragmatic legitimacy, the preceding sections demonstrated that DPR has the potential to extend the southern Nevada water supply (DPR 2) or potentially provide a higher‐quality drinking water (DPR 1). The original hypothesis was that DPR could also yield a net reduction in energy consumption by reducing or eliminating the pumping requirements for the RFCs. Figure 7 summarizes the differences in annual energy cost and life cycle unit energy costs for each of the scenarios. Again, it is important to note that the energy costs refer only to pumping and treatment of the RFC and DPR portions of the water supply as it was assumed that costs associated with other water supplies (e.g., baseline Colorado River allocation) would be similar between the various scenarios.

For raw water augmentation, which involved pumping purified water to the RMWTF, the annual energy costs for both DPR treatment trains exceeded the RFC scenarios. For DPR 1, this was partly due to the energy intensive nature of the treatment train as indicated by the life cycle unit energy cost of more than $58/AF. DPR 2 was comparable to the RFC scenarios, with a life cycle unit energy cost of ~$42/AF. The other issue for DPR with raw water augmentation was the significant cost of pumping water from Las Vegas Wash to the AWPF and then to the RMWTF for additional treatment. These pumping costs accounted for 51% and 64% of the overall DPR energy cost (i.e., DPR pumping + DPR treatment) for DPR 1 and DPR 2, respectively. For finished water augmentation (i.e., direct distribution), the energy costs decreased significantly, with both treatment trains offering an economic advantage over the RFC scenarios (Figure 7). In fact, the life cycle unit energy costs decreased to $31/AF and $20/AF for DPR 1 and DPR 2, respectively. These reductions amounted to a net present savings of more than $150 million and $250 million in 2015 U.S. dollars (Figure S8).

### 3.4 NPW evaluation

To understand the full economic implications of each scenario, a NPW analysis was warranted (Table 1). The NPW for the RFC scenarios included the energy costs for pumping and treating the RFCs at RMWTF and the capital cost for the Nevada Groundwater Development Project (when applicable). The NPW for the DPR scenarios included energy costs for pumping feed water from the Las Vegas Wash to the AWPF, energy costs for pumping and treating RFCs and DPR product water (when applicable) at RMWTF, the phase 1 and phase 2 capital costs for the DPR treatment train and DPR pipeline, and overall O&M costs for the operation of the DPR treatment train.

The baseline RFC scenarios were each ~$0.6 billion overall and ~$45/AF, while the groundwater pipeline scenario was considerably higher at $2.8 billion and $225/AF. For raw water augmentation, the DPR 1 scenarios ranged from $1.6 to $4.0 billion ($134–$338/AF overall [RFC + DPR] or $328–$391/AF for DPR flows only), and the DPR 2 scenarios ranged from $1.1 to $2.1 billion ($85–$155/AF overall or $155–$190/AF of DPR). Although the DPR scenarios with direct distribution (i.e., finished water augmentation) achieved a net savings in energy costs (Figure S7), the NPWs were still considerably higher than the baseline RFC scenarios after accounting for the capital and overall O&M costs. The DPR 1 scenarios ranged from $1.6 to $3.8 billion ($128–$320/AF overall or $310–$370/AF of DPR), and the DPR 2 scenarios ranged from $1.0 to $1.8 billion ($79–$137/AF overall or $137–$169/AF of DPR).

When focusing only on overall costs, DPR does not appear to be an attractive alternative in Las Vegas, in part because the area is well positioned to leverage its current IPR (or RFC) configuration. Cooley and Wilkinson (2012) reported energy cost estimates for conventional drinking water treatment ranging from $2/AF (25th percentile) to $33/AF (75th percentile). Because pumping from Lake Mead ($36/AF) constitutes such a large percentage of the cost of Las Vegas drinking water, treatment at $33/AF would increase the NPW from $45/AF to $60/AF in the RFC scenario—still considerably cheaper than DPR 1 and DPR 2. Additional capital investments for the RFC scenario, changes in the energy price structure (currently at $0.05/kWh), or realization of other externalities (Kiparsky et al., 2013) would be necessary to increase the attractiveness of DPR.

### 3.5 Economic impacts of environmental discharges and emissions

**Figure 8:**
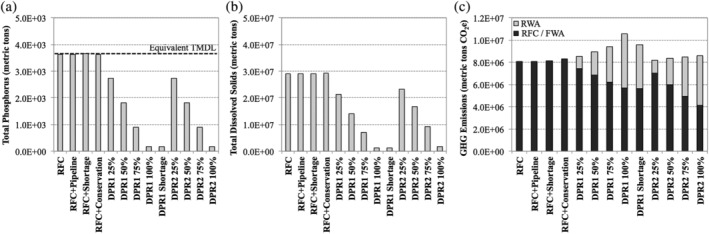
Summary of (a) total phosphorus loadings to Lake Mead, (b) total dissolved solids loadings to Lake Mead, and (c) greenhouse gas emissions over the entire study period (2015–2065). In (a), the equivalent total maximum daily load (TMDL) represents the total phosphorus loading to Lake Mead assuming continuous TMDL compliance throughout the year. In (c), RWA refers to DPR with raw water augmentation, FWA refers to DPR with finished water augmentation (i.e., direct distribution), and RFC refers to the status quo return flow credits approach. DPR, direct potable reuse; DPR 1, DPR treatment train 1; DPR 2, DPR treatment train 2; RFC, return flow credit

This study also quantified potential discharges and emissions of TP, TDS, and GHGs. Figure 8a shows the TP loadings to Lake Mead via the Las Vegas Wash for the different water supply scenarios. The RFC scenarios all resulted in 3,700 metric tons of TP being discharged to Lake Mead because the model forced adjustments in the wastewater effluent TP concentration to maintain compliance with the TMDL at all times. For the status quo RFC scenario, this resulted in maximum allowable wastewater effluent TP concentrations of 0.23 mg/L in 2015 and 0.15 mg/L toward the end of the study period (Figure S7). The DPR scenarios reduced TP loadings by 910–3,500 metric tons of TP, depending on the diversion percentage. Therefore, DPR could theoretically allow for higher wastewater effluent TP concentrations, which could reduce costs associated with chemical and biological phosphorus removal at the conventional WWTPs. These savings were not quantified in this study, and current and future TP compliance costs are not a significant concern for local wastewater agencies.

In addition to the aesthetic and regulatory implications of high TDS concentrations in drinking water, salinity can also have detrimental economic impacts in other contexts (e.g., reductions in agricultural yields). Adverse impacts along the Colorado River have been described on a concentration basis ($30,000–$300,000/mg/L; Anderson & Kleinman, 1978), a loading basis ($125/metric ton; Borda, 2004; Lohman, Millike, Dorn, & Tuccy, 1988), and an overall basis ($300–$500 million annually; Venkatesan et al., 2011b). Assuming a TDS “cost” of $125/metric ton, the reduction in TDS achieved by DPR 1 with 100% diversion (Figure 8b) amounts to an economic benefit of $1.7 billion in 2015 U.S. dollars over the study period. Based on the information in Table 1, this would reduce the NPW of the DPR 1 scenario with 100% diversion to $2.1–$2.3 billion, although this is still considerably higher than the baseline RFC scenario (~$0.6 billion). It is also currently unclear how these TDS “savings” could actually be realized by the agency incurring the DPR costs. In the future, a salinity trading framework among Colorado River stakeholders (e.g., to maintain treaty commitments with Mexico) might provide a formal basis for monetizing TDS credits.

The GHG emission trends for the various water supply scenarios (Figure 8c) were consistent with the energy costs described earlier. GHG emission rates remained constant at ~650 kg CO_2e_/AF for the RFC scenarios and decreased to as low as 477 kg CO_2e_/AF and 309 kg CO_2e_/AF for finished water augmentation with DPR 1 and DPR 2, respectively. For comparison, Raucher and Tchobanoglous (2014) reported a carbon footprint of 373 kg CO_2e_/AF for a treatment train comparable to DPR 1. GHG emissions have also been monetized, including markets for allowance trading or more explicit carbon taxes (Luckow et al., 2015). Beyond the implications for climate change, these frameworks can be used to characterize GHG emissions in an economic context. Using the CO_2e_ price structure described earlier (Luckow et al., 2015), the reduction in GHG emissions achieved by DPR amounts to a relatively minor economic benefit of up to $68 million for DPR 1 and $114 million for DPR 2 (in 2015 U.S. dollars) over the study period.

## 4 CONCLUSIONS

This study highlighted some of the limitations of Las Vegas' water supply framework that could be mitigated through DPR implementation, but it also identified limitations that must be overcome to make centralized DPR a viable option for the region. Las Vegas is uniquely suited for IPR because of its ability to discharge treated wastewater upstream of its drinking water intakes while achieving high dilution ratios in Lake Mead (~100:1). As a result, local water policies often focus on reducing consumptive use through outdoor conservation to maximize RFCs. Although the elevation of Lake Mead has declined to historically low levels, the tremendous energy required to recapture these return flow credits has been mitigated by exceptionally low energy costs ($0.05/kWh). There are currently few regulatory drivers to address potentially problematic water quality (e.g., TDS) or environmental health concerns (e.g., GHG emissions), and with current drought conditions, any water returned to Lake Mead is perceived positively. Therefore, any significant water policy changes would require a clear economic advantage to overcome the cognitive legitimacy of the status quo approach. Nevertheless, this study demonstrated that conservation and other strategic water supply measures are necessary to ensure a reliable water supply for Las Vegas in the future. Although beyond the scope of the current study, decentralized DPR might become more attractive as Las Vegas expands geographically, thereby warranting future evaluations.

The attractiveness of DPR might be entirely different in other cities, particularly if certain metrics are weighted differently or capital investments are required for implementation of IPR or for continuation of status quo water supply alternatives. For example, potable reuse is an increasingly attractive option in California where costs associated with imported water continue to rise. DPR with RO‐based treatment trains can obviously achieve a high‐quality drinking water, but there are clear hurdles that must be overcome. For Las Vegas, centralized DPR with RO and direct distribution could reduce energy consumption, GHG emissions, and contaminant loadings to Lake Mead, but the NPW was cost prohibitive. In addition, due to losses associated with brine disposal, RO‐based DPR exacerbated Las Vegas' future water supply deficit. DPR with O_3_‐biofiltration was significantly cheaper but may only be feasible in areas with low TDS (and possibly bulk organic matter) concentrations in local source waters.

Potable reuse is highly site‐specific, with varying regulatory frameworks, levels of public acceptance, and practical considerations impacting its implementation in different cities. Even though centralized DPR may not be a viable alternative for southern Nevada given its ability to efficiently leverage its RFCs, this research presented a conceptual‐level framework for evaluating potable reuse alternatives in other regions. In locations with higher energy costs and greater elevation changes, the energy cost differential coupled with a cost‐efficient DPR treatment train may yield an economically viable alternative. Therefore, it is advisable for all municipalities in water‐stressed regions to evaluate the sustainability of DPR across a broad range of criteria (e.g., through a full triple bottom line analysis), particularly as DPR gains cognitive legitimacy over time.

## Supporting information

Supporting Information
