# Supplementary material for: Evaluating the sustainability of indirect potable reuse and direct potable reuse: a southern Nevada case study
Source: AWWA Water Sci. 2019 Aug 27;1(4):e1153. doi: 10.1002/aws2.1153 (PMC6851734; doi:10.1002/aws2.1153)
Supplement: Supplementary file 1 — Supporting Information [file AWS2-1-na-s001.pdf]

Electronic Supplementary Information

Evaluating the Sustainability of IPR and DPR:

A Southern Nevada Case Study

CORY DOW[1,2], SAJJAD AHMAD[1], KRYSTYNA STAVE[3], DANIEL GERRITY[1,4*]

[1]*Department of Civil and Environmental Engineering, University of Nevada, Las Vegas, Box 454015, 4505 S. Maryland Parkway, Las Vegas, NV 89154-4015, United States*

[2]*Carollo Engineers, 376 E. Warm Springs Rd. #250, Las Vegas, NV 89119, United States*

[3]*School of Public Policy and Leadership, University of Nevada, Las Vegas, Box 454030, 4505 S. Maryland Parkway, Las Vegas, NV 89154-4030, United States*

[4]*Applied Research and Development Center, Southern Nevada Water Authority, P.O. Box 99954, Las Vegas, NV 89193, United States*

[*]Corresponding author. P: (702) 856-3518. F: (702) 856-3647. Email: Daniel.Gerrity@unlv.edu; Daniel.Gerrity@snwa.com.

Submitted for publication to AWWA Water Science

Text S1. Summary of equations and assumptions

Population forecasting: from Tra (2015)

$$\text{Population} = (-1.864\times10^{2}) \times (\text{Year})^{2} + (7.849\times10^{5}) \times (\text{Year}) - 8.223\times10^{8} \quad \text{(Eq. S1)}$$

where, Year = calendar year.

Pumping energy:

$$\text{EHP} = \frac{Q\times H}{3957\times E_p\times E_m} \quad \text{(Eq. S2)}$$

where, EHP = electrical horsepower

Q = flow (gallons/minute)

H = elevation difference (feet)

3957 = conversion factor for electrical horsepower

(33000 ft-lb/min divided by 8.34 lb/gallon)

E_p = pump efficiency (assumed to be 0.80)

E_m = motor efficiency (assumed to be 0.92)

$$\text{Energy} = \text{EHP} \times 0.746 \times 24 \times 365 \quad \text{(Eq. S3)}$$

where, Energy = annual pumping energy consumption (kWh/year)

0.746 = conversion from EHP to kW

Advanced Water Purification Facility Energy Consumption:

Typical and assumed values for energy consumption in the DPR 1 and DPR 2 treatment trains are summarized in Table S1.

Table S1. Typical and assumed energy consumption values for the AWPF

Treatment Technology	Typical[1] kWh/AF	Assumed kWh/AF	DPR 1	DPR 2
MF/UF	240-360	295[2,3]	✓	✓
RO	550-700	504[2,3]	✓	
UV AOP	98-326	85[2,3]	✓	✓
O_3	N/A	128[2]		✓
BAC	N/A	31[2]		✓

[1]Raucher and Tchobanoglous (2014)
[2]Gerrity et al. (2014)
[3]Based on full-scale data from California

Greenhouse gas emissions:

Nearly 88% of the energy consumed in Nevada comes from outside the state and is primarily sourced from natural gas (EIA, 2019). However, Nevada generates the most geothermal energy in the U.S., amounting to 10% of the state's energy supply, and solar energy amounts to approximately 6%. Overall, renewable energy accounts for 22% of the state's energy supply. Based on historical emissions in 2015, Nevada's energy portfolio resulted in a carbon intensity [or greenhouse gas (GHG) emission rate] of 0.38 kg of CO_{2e} per kWh (or 0.83 lb CO_{2e} per kWh) (EIA, 2017).

Capital and O&M costs:

Capital (Table S2) and O&M (Table S3) cost estimates for the DPR treatment trains were developed using a conceptual-level (Class 4) approach with an estimated accuracy of -30% to +50% (AACE, 2011; Plumlee et al., 2014). The Plumlee et al. (2014) unit cost curves were based on 2011 U.S. dollars, which required adjustments to 2015 U.S. dollars using an appropriate Construction Cost Index (CCI) (ENR, 2018). The relevant CCIs are noted below Tables S2 and S3. For the finished water augmentation cost estimates, an engineered storage buffer (ESB) with an 8-hr response retention time was included for both DPR 1 and DPR 2. The cost associated with the ESB was estimated at $1.25 per gallon of storage (Tchobanoglous et al., 2015), and the

storage volume was calculated based on the target response retention time and the design flow rates for phase 1 construction and the phase 2 expansion. For the O&M costs for DPR 1, a cost of $155/AF was included for brine disposal via evaporation ponds (Raucher and Tchobanoglous, 2014). Finally, the Plumlee et al. (2014) unit cost curves for O&M were based on an electricity cost of $0.0988/kWh. Table S3 summarizes modified unit cost curves developed as part of the current study to account for SNWA's electricity cost of $0.05/kWh. The modified curves were developed using the raw data from Plumlee et al. (2014) but with modified energy costs.

Table S2. Capital cost estimate: Unit cost curves from Plumlee et al. (2014)

Technology	Capital Cost ($M/MGD)[a,b]	DPR 1	DPR 2
MF/UF	3.57 × (Plant Capacity, in MGD)$^{-0.22}$	✓	✓
RO	7.14 × (Plant Capacity, in MGD)$^{-0.22}$	✓	
UV AOP	0.474 × (Plant Capacity, in MGD)$^{-0.056}$	✓	✓
O_3	2.26 × (Plant Capacity, in MGD)$^{-0.54}$		✓
O_3 Dose Adjustment[c]	0.0294 × (Plant Capacity, in MGD) × (r – 1)		✓
BAC (20 min EBCT)	1.52 × (Plant Capacity, in MGD)$^{-0.15}$		✓

[a]Based on 2011 U.S. dollars
[b]$1.25/gallon added for ESB with 8-hr response retention time for finished water augmentation (see Table S6)
[c]Error in Plumlee et al. (2014): should be 0.0294 instead of 0.0156; r = (target O_3 dose, in mg/L)/(3.0 mg/L)

- ENR CCI for September 2011 = 9116
- ENR CCI for September 2015 = 10065

Table S3. Annual O&M cost estimate: Modified unit cost curves from Plumlee et al. (2014)

Technology	O&M Cost ($M/MGD)[a]	DPR 1	DPR 2
MF/UF	0.29 × (Plant Capacity, in MGD)$^{-0.22}$	✓	✓
RO[b]	0.35 × (Plant Capacity, in MGD)$^{-0.16}$	✓	
Membrane Redundancy	-0.20 × (Plant Capacity, in MGD)$^{-0.83}$	✓	
UV AOP	0.028 × (Plant Capacity, in MGD)$^{-0.029}$	✓	✓
O_3	0.0035 × (Plant Capacity, in MGD)$^{-0.052}$		✓
O_3 Dose Adjustment	0.0025 × (Plant Capacity, in MGD) × (r – 1)		✓
BAC (20 min EBCT)	0.026 × (Plant Capacity, in MGD)$^{-0.051}$		✓

[a]Based on 2011 U.S. dollars
[b]$155/AF added for brine disposal via evaporation ponds (Raucher and Tchobanoglous, 2014)

- ENR CCI for September 2011 = 9116
- ENR CCI for September 2015 = 10065

Capital costs associated with construction of the DPR pipeline were developed using the Carollo Cost Estimating System (CCES). The CCES uses Carollo Engineers' proprietary

database that accounts for material, equipment, and labor costs for recent design projects. Summaries of the estimated construction costs (in 2017 U.S. dollars) are provided in Tables S4 and S5. A contingency of 30% and contractor overhead and profit of 15% were added to the subtotal to generate the overall total cost estimate.

Table S4. DPR pipeline for raw water augmentation

Scenario	Q_{design} m^3/s	Diameter m	v_{max} m/s	Length km	Earthwork[a] ($M)	Pipe/Fittings[a] ($M)	Subtotal[a] ($M)	Total[a] ($M)
DPR 1 25%	2.87	1.52	1.75	9.22	$5.9	$16.0	$21.9	$31.8
DPR 1 50%	5.73	2.13	1.78	9.22	$7.6	$23.6	$31.2	$45.3
DPR 1 75%	8.55	2.74	1.61	9.22	$9.3	$31.6	$40.9	$59.3
DPR 1 100%	10.9	3.05	1.67	9.22	$10.2	$35.7	$45.9	$66.6
DPR 1 Shortage	10.6	2.74	1.79	9.22	$9.3	$31.6	$40.9	$59.3
DPR 2 25%	3.19	1.52	1.94	9.22	$5.9	$16.0	$21.9	$31.8
DPR 2 50%	6.38	2.13	1.98	9.22	$7.6	$23.6	$31.2	$45.3
DPR 2 75%	9.57	2.74	1.80	9.22	$9.3	$31.6	$40.9	$59.3
DPR 2 100%	12.3	3.05	1.87	9.22	$10.2	$35.7	$45.9	$66.6

[a]Based on 2017 U.S. dollars

- ENR CCI for September 2015 = 10065
- ENR CCI for January 2017 = 10532

Table S5. DPR pipeline for finished water augmentation (i.e., direct distribution)

Scenario	Q_{design} m^3/s	Diameter m	v_{max} m/s	Length km	Earthwork[a] ($M)	Pipe/Fittings[a] ($M)	Subtotal[a] ($M)	Total[a] ($M)
DPR 1 25%	2.87	1.52	1.75	2.31	$1.5	$4.0	$5.5	$7.9
DPR 1 50%	5.73	2.13	1.78	2.31	$1.9	$5.9	$7.8	$11.3
DPR 1 75%	8.55	2.74	1.61	2.31	$2.3	$7.9	$10.2	$14.8
DPR 1 100%	10.9	3.05	1.67	2.31	$2.5	$8.9	$11.5	$16.6
DPR 1 Shortage	10.6	2.74	1.79	2.31	$2.3	$7.9	$10.2	$14.8
DPR 2 25%	3.19	1.52	1.94	2.31	$1.5	$4.0	$5.5	$7.9
DPR 2 50%	6.38	2.13	1.98	2.31	$1.9	$5.9	$7.8	$11.3
DPR 2 75%	9.57	2.74	1.80	2.31	$2.3	$7.9	$10.2	$14.8
DPR 2 100%	12.3	3.05	1.87	2.31	$2.5	$8.9	$11.5	$16.6

[a]Based on 2017 U.S. dollars

- ENR CCI for September 2015 = 10065
- ENR CCI for January 2017 = 10532

Table S6 provides a summary of engineered storage buffer capacity for the various finished water augmentation (i.e., direct distribution) scenarios. Sizing of the engineered storage buffer was based on an 8-hr response retention time and the phase 1 (2035) and phase 2 (buildout) design flows (Figure S1).

Table S6. Engineered storage buffer capacity [in megaliters (ML) and million gallons (MG)] for finished water augmentation (i.e., direct distribution) scenarios

Scenario	Phase 1 ML	Phase 2 ML	Total ML	Phase 1 MG	Phase 2 MG	Total MG
DPR 1 25%	67	16	83	18	4	22
DPR 1 50%	133	32	165	35	8	44
DPR 1 75%	200	46	246	53	12	65
DPR 1 100%	255	60	315	67	16	83
DPR 1 Shortage	255	51	305	67	13	81
DPR 2 25%	74	18	92	20	5	24
DPR 2 50%	148	36	184	39	10	49
DPR 2 75%	222	53	276	59	14	73
DPR 2 100%	283	71	354	75	19	94

The Nevada Groundwater Development Project pipeline had a projected capital cost of $3.2 billion in 2007 U.S. dollars (ENR CCI for September 2007 = 8050), which results in an estimated capital cost of $4.0 billion in 2015 U.S. dollars (ENR CCI for September 2015 = 10065). Based on model output identifying a need for imported groundwater, capital costs for construction of the pipeline were incurred in 2035.

Treatment costs associated with local groundwater and new surface water withdrawals were assumed to be identical between systems. Therefore, operational costs for drinking water treatment were applied only to return flow credits and DPR flows in the raw water augmentation scenario. These costs were based on Cooley and Wilkinson (2012), which summarized data for 92 drinking water utilities in the United States. The 25th percentile energy intensity for high flow drinking water systems was adopted for the current study (120 kWh per million gallons or $6.00/MG based on $0.05/kWh for SNWA). This baseline energy cost was augmented with $5.11/MG for ozone production—for a total energy cost = $11.11/MG—and $16.28/MG for chemical costs (Giltner, 2019). Treatment costs for local and imported groundwater were assumed to be negligible (Cooley and Wilkinson, 2012).

Figure S1. Summary of DPR product water flow rates for the advanced water purification facility in the (left) DPR 1 and (right) DPR 2 scenarios. Flow rates differ because of water losses in DPR 1 due to reverse osmosis brine disposal.

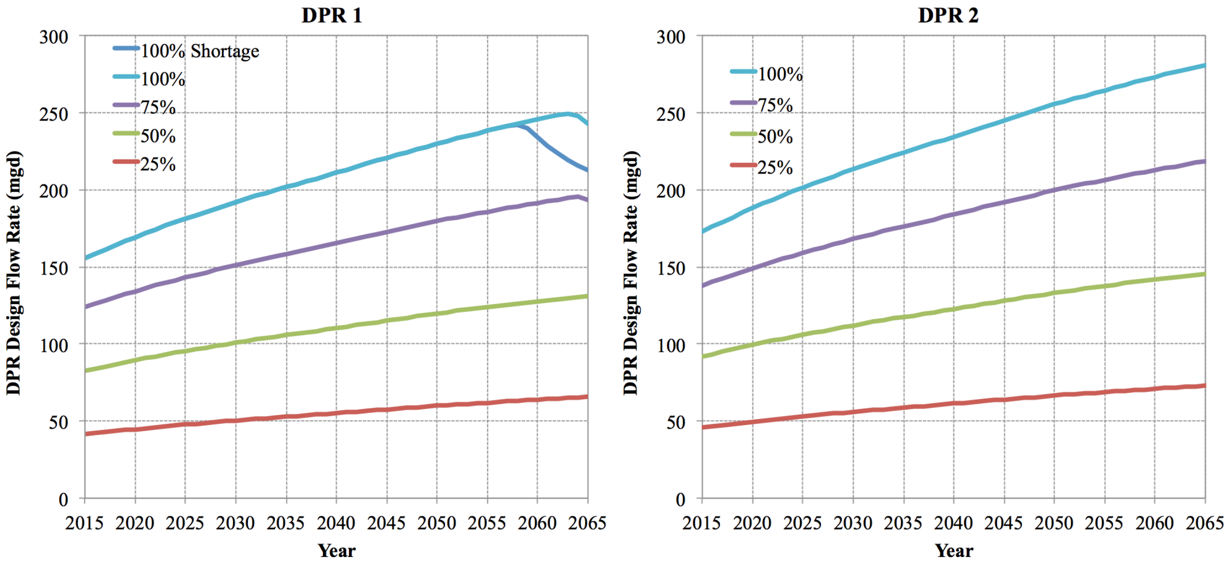

Figure S2. System dynamics model structure in STELLA 10.1 platform: Water flow sector.

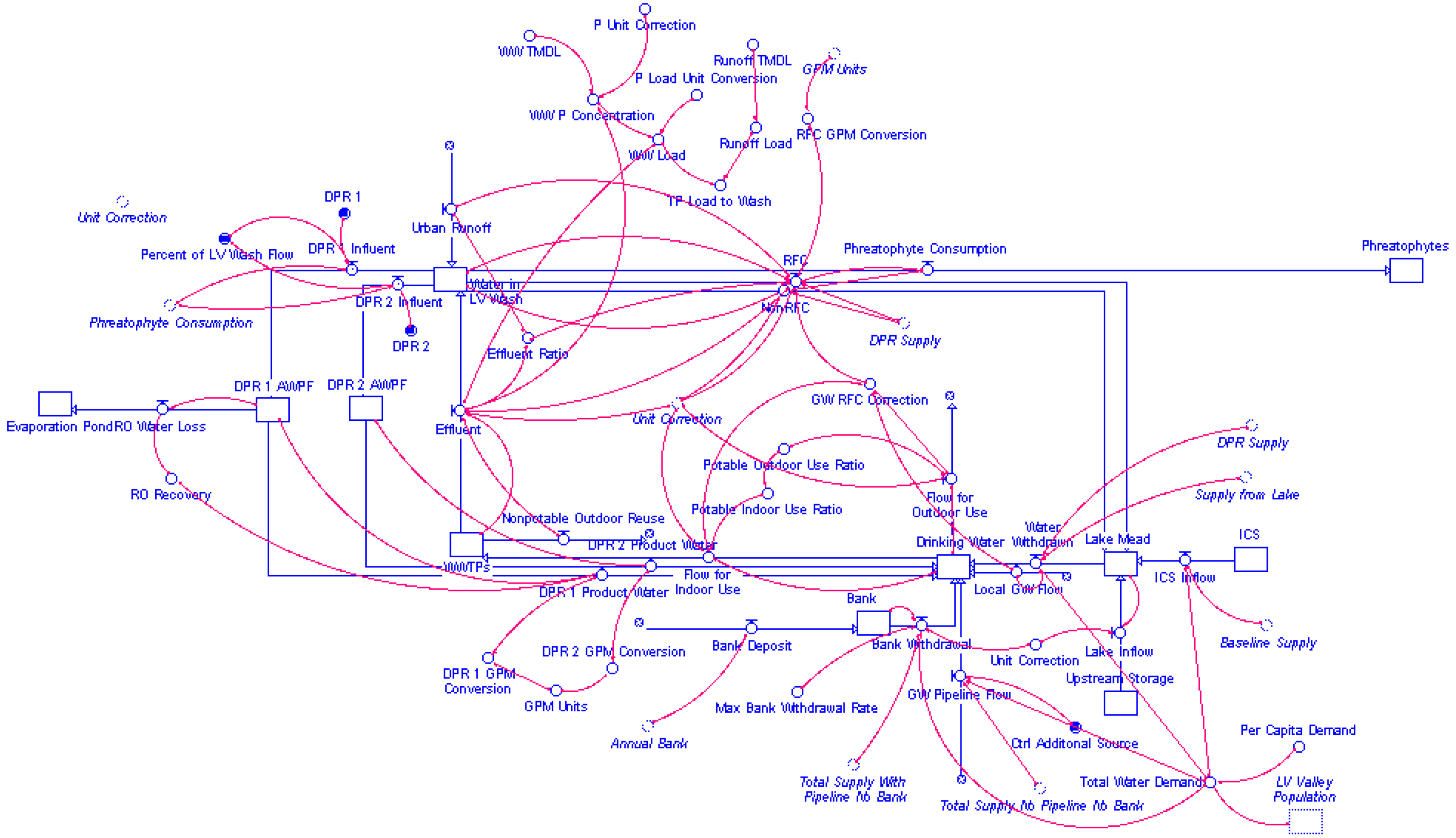

Figure S3. System dynamics model structure in STELLA 10.1 platform: Total dissolved solids sector.

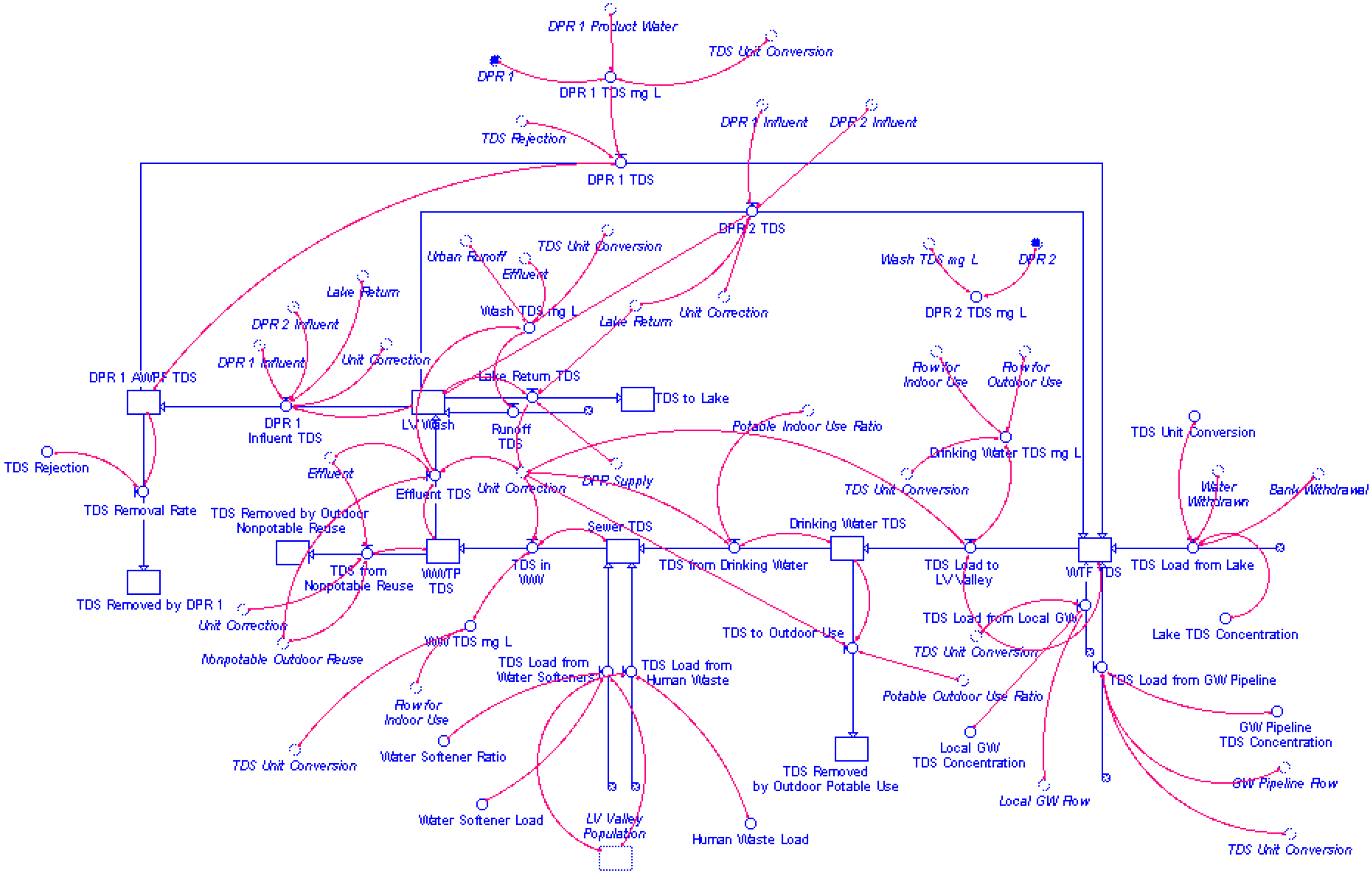

Figure S4. System dynamics model structure in STELLA 10.1 platform: Water supply and elevation sector.

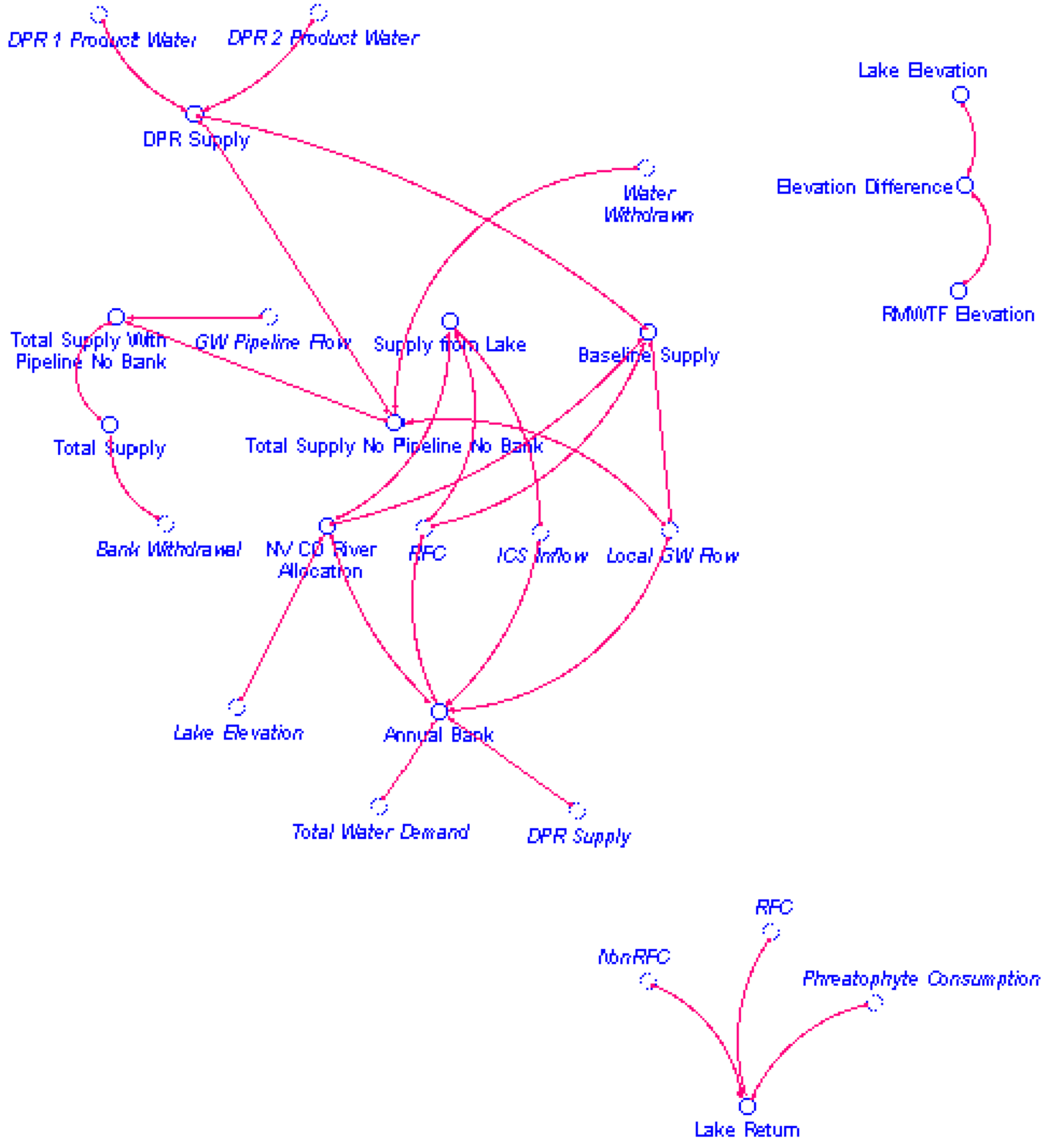

Figure S5. System dynamics model structure in STELLA 10.1 platform: (top) Energy consumption sector and (bottom) greenhouse gas emission sector.

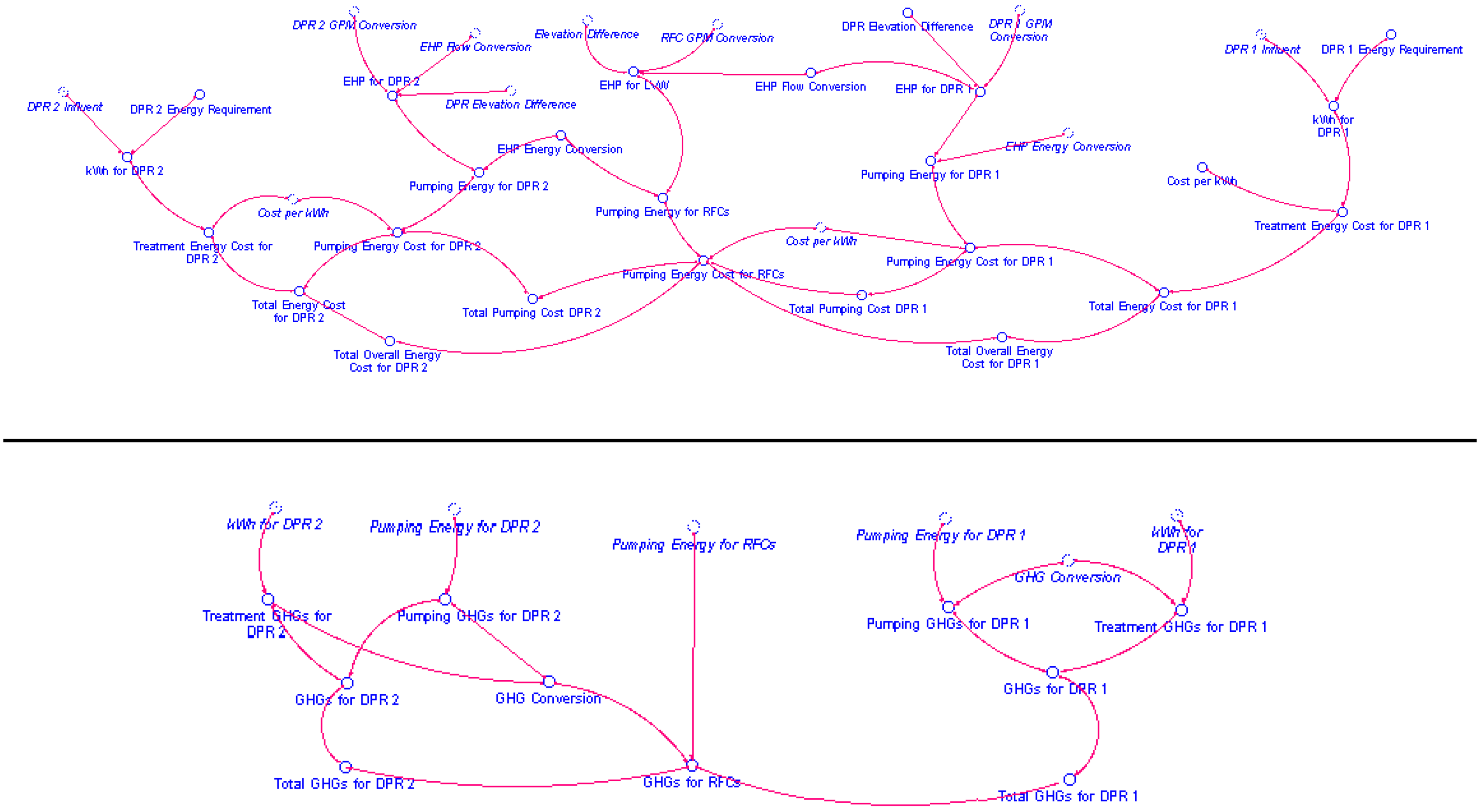

Figure S6. System dynamics model structure in STELLA 10.1 platform: (left) Population and (right) phosphorus sectors.

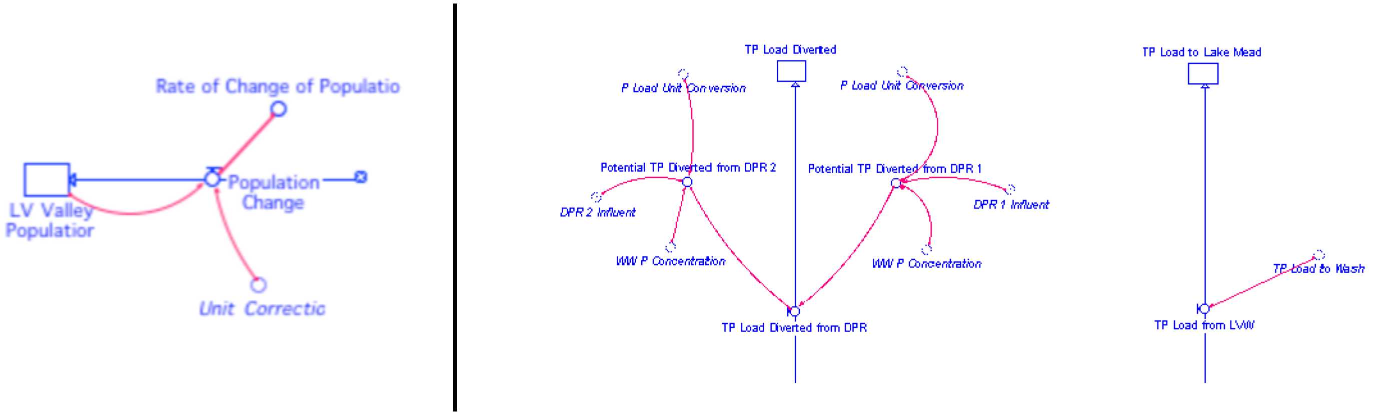

Figure S7. Summary of total dissolved solids (TDS) and total phosphorus (TP) concentrations for the baseline return flow credits (RFC) scenario.

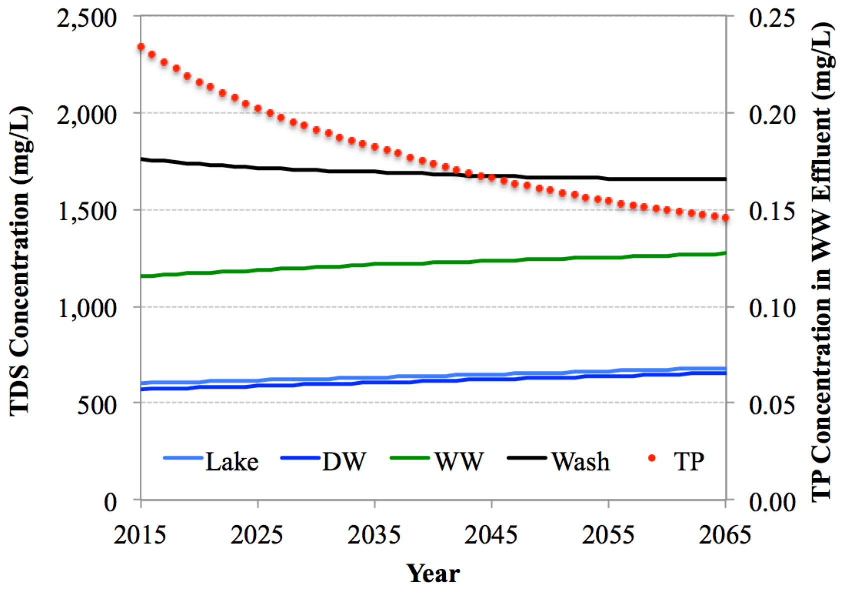

Figure S8. Net present energy cost savings calculated relative to the status quo return flow credits (RFC) scenario.

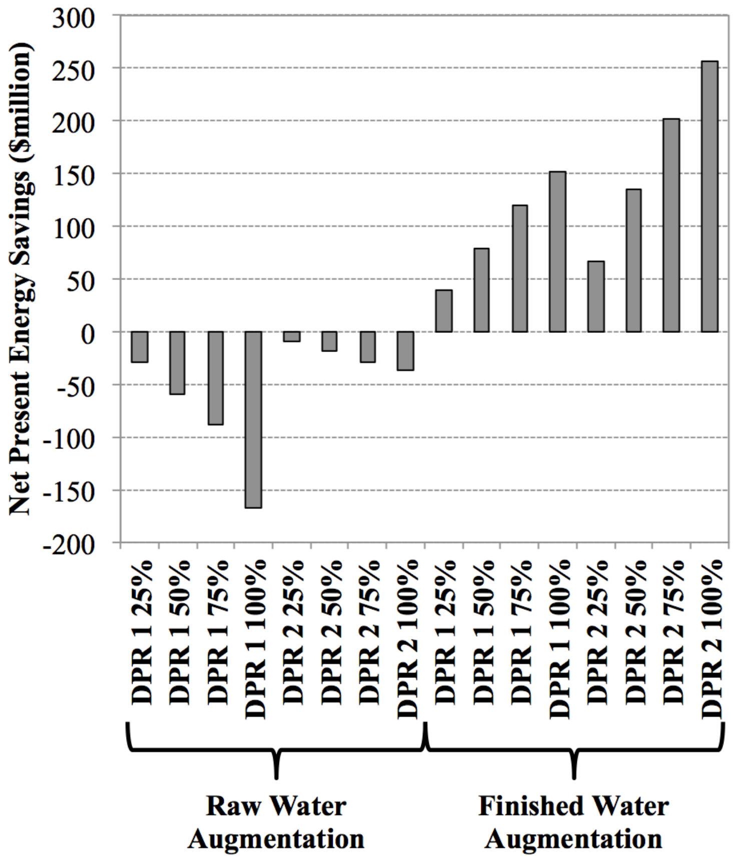

References

AACE (2011). Cost Estimate Classification System. Recommended Practice R17-97. AACE International.

Cooley, H., Wilkinson, R. (2012). Implications for future water supply sources for energy demands. WateReuse Research Foundation. Alexandria, VA.

EIA (2017). Nevada Electricity Profile 2015. United States Energy Information Administration. https://www.eia.gov/electricity/state/archive/2015/nevada/index.php. Accessed: June 27, 2019.

EIA (2019). Technical notes and documentation. United States Energy Information Administration. https://www.eia.gov/state/seds/seds-technical-notes-complete.php?sid=NV. Accessed: July 2, 2019.

ENR (2018). Construction Cost Index History. Engineering News-Record. https://www.enr.com/economics/historical_indices. Accessed: May 31, 2018.

Gerrity, D., Owens-Bennett, E., Venezia, T., Stanford, B.D., Plumlee, M.H., Debroux, J., Trussell, R.S. (2014). Applicability of ozone and biological activated carbon for potable reuse. Ozone Sci. Eng. 36, 123-137.

Giltner, R. (2019). Personal email communication with the Director of Water Quality and Treatment at the Southern Nevada Water Authority. June 25, 2019.

Plumlee, M.H., Stanford, B.D., Debroux, J., Hopkins, D.C., Snyder, S.A. (2014). Costs of advanced treatment in water reclamation. Ozone Sci. Eng. 36, 485-495.

Raucher, R.S., Tchobanoglous, G. (2014). The opportunities and economics of direct potable reuse. WateReuse Research Foundation. Alexandria, VA.

Tchobanoglous, G., Cotruvo, J., Crook, J., McDonald, E., Olivieri, A., Salveson, A., Trussell, R.S. (2015). Framework for direct potable reuse. WateReuse Research Foundation. Alexandria, VA.

Tra, C.I. (2015). Population forecasts: Long-term projections for Clark County, Nevada 2015-2050. Center for Business and Economic Research. http://www.clarkcountynv.gov/comprehensive-planning/demographics/Documents/2015_Population_Forecasts.pdf. Accessed: May 31, 2018.
